# Dissection of jasmonate functions in tomato stamen development by transcriptome and metabolome analyses

**DOI:** 10.1186/s12915-015-0135-3

**Published:** 2015-04-21

**Authors:** Susanne Dobritzsch, Martin Weyhe, Ramona Schubert, Julian Dindas, Gerd Hause, Joachim Kopka, Bettina Hause

**Affiliations:** Leibniz Institute of Plant Biochemistry, Weinberg 3, D06120 Halle, Germany; Martin Luther University Halle Wittenberg, Biocenter, Electron Microscopy, Weinbergweg 22, D06120 Halle, Germany; Max Planck Institute of Molecular Plant Physiology, Am Muehlenberg 1, D14476 Potsdam, (OT) Golm, Germany; Present address: Department of Botany I, University of Würzburg, Julius-von-Sachs-Platz 2, D97082 Würzburg, Germany

**Keywords:** Array hybridization, Desiccation, Ethylene, Flower development, Jasmonic acid, Jasmonic acid-insensitive, Metabolite profiling, Pollen development

## Abstract

**Background:**

Jasmonates are well known plant signaling components required for stress responses and development. A prominent feature of jasmonate biosynthesis or signaling mutants is the loss of fertility. In contrast to the male sterile phenotype of *Arabidopsis* mutants, the tomato mutant *jai1-1* exhibits female sterility with additional severe effects on stamen and pollen development. Its senescence phenotype suggests a function of jasmonates in regulation of processes known to be mediated by ethylene. To test the hypothesis that ethylene involved in tomato stamen development is regulated by jasmonates, a temporal profiling of hormone content, transcriptome and metabolome of tomato stamens was performed using wild type and *jai1-1*.

**Results:**

Wild type stamens showed a transient increase of jasmonates that is absent in *jai1-1*. Comparative transcriptome analyses revealed a diminished expression of genes involved in pollen nutrition at early developmental stages of *jai1-1* stamens, but an enhanced expression of ethylene-related genes at late developmental stages. This finding coincides with an early increase of the ethylene precursor 1-aminocyclopropane-1-carboxylic acid (ACC) in *jai1-1* and a premature pollen release from stamens, a phenotype similarly visible in an ethylene overproducing mutant. Application of jasmonates to flowers of transgenic plants affected in jasmonate biosynthesis diminished expression of ethylene-related genes, whereas the double mutant *jai1-1 NeverRipe* (ethylene insensitive) showed a complementation of *jai1-1* phenotype in terms of dehiscence and pollen release.

**Conclusions:**

Our data suggest an essential role of jasmonates in the temporal inhibition of ethylene production to prevent premature desiccation of stamens and to ensure proper timing in flower development.

**Electronic supplementary material:**

The online version of this article (doi:10.1186/s12915-015-0135-3) contains supplementary material, which is available to authorized users.

## Background

Jasmonic acid (JA) and its metabolites, such as its methyl ester (JAME) or amino acid conjugates, all of them commonly named jasmonates, are ubiquitously occurring signaling compounds in plants and are formed in response to biotic and abiotic stress as well as in development [1]. Jasmonates are lipid-derived compounds synthesized from α-linolenic acid released from plastid membranes and are synthesized by one of seven different branches of the lipoxygenase (LOX) pathway [2,3]. LOX and the two following enzymatic steps are located in the plastids and involve the action of an ALLENE OXIDE SYNTHASE and an ALLENE OXIDE CYCLASE (AOC) leading to formation of the intermediate cyclopentenone *cis*-12-oxo-phytodienoic acid (OPDA). Further reactions occur in peroxisomes and include the OPDA REDUCTASE 3 (OPR3) and three cycles of fatty acid β-oxidation. Within the JA pathway the AOC-catalyzed step is regarded as the crucial step as here the exclusive formation of the enantiomeric form occurring in natural cyclopentanones like JA is facilitated [4,5]. JA can be enzymatically converted into numerous conjugates and derivatives, some of which are biologically active, such as JAME [6], *cis*-jasmone [7,8] and JA-amino acid conjugates [8,9]. Among the latter, (+)-*7-iso*-jasmonoyl isoleucine (JA-Ile) was demonstrated to be the biologically active form of jasmonates by mediating binding of the co-receptor proteins CORONATINE INSENSITIVE1 (COI1) and JASMONATE ZIM DOMAIN (JAZ) [10-13].

JA-Ile is involved in most JA-dependent processes mediated by the F-box protein COI1 in a proteasome-dependent manner [1]. As evidenced by phenotypic analyses of *Arabidopsis* mutants, jasmonates are involved in various developmental processes, one of them being flower development. The coronatine- and jasmonate-insensitive mutant *coi1* [14] as well as the JA biosynthesis mutants *fad3-2fad7-2fad8* [15], *dad1* [16], *opr3* [17], and *dde1* [18] cannot produce viable pollen. All of these mutants exhibit an identical male-sterile phenotype: (1) anther filaments do not elongate sufficiently to position the locules above the stigma at anthesis, (2) anthers lack the proper dehiscence of the stomium at the time of flower opening, and (3) pollen is produced in a smaller amount than in the wild type and does not germinate [19,20]. Combining these phenotypic features with the fact that mutants defective in JA perception and/or synthesis have much less water loss in anther tissue than wild type plants, JA has been thought to control dehiscence by regulating water transport into the filament and out of the anthers of *Arabidopsis* [16,21]. Moreover, it has been assumed that JA is required for the expression of genes involved in water transport in anthers [16].

Similar to the *Arabidopsis* mutant *coi1*, a mutant impaired in the tomato ortholog of *COI1* was isolated in a genetic screen for plants that are unable to accumulate defense-related proteins in response to JAME application [22]. This mutant, called *jasmonic acid-insensitive1-1* (*jai1-1*) and identified in a screen of a fast-neutron-mutagenized population of Micro-Tom plants, exhibits a 6.2-kb deletion in *SlCOI1*, is deficient in all JA-Ile mediated responses and fails to set seeds [23]. Reciprocal crosses revealed that *jai1-1* plants are male fertile, but female sterile [22]. This is in agreement with data showing that not only JA perception but also JA formation is required for female development: AOC protein occurs specifically in ovules, and OPDA, JA, and JA-Ile accumulate preferentially in the ovary of flower buds, where the levels markedly exceed those detected in non-stressed leaves [24]. The organ-specific accumulation of JA and – most importantly – of JA-Ile may result in organ-specific regulation of gene expression. Indeed, a number of JA-induced genes are specifically expressed within ovules [24], but their regulation by JA in gametophytic organs has not yet been proven.

The ability of *jai1-1* pollen to induce normal seed set when crossed to a wild type pistillate flower indicates that JA perception and downstream signaling events might not be essential for the production of viable pollen in tomato [23]. However, *jai1-1* plants also exhibit defects in male reproductive function, such as a reduction in pollen viability and germination, tissue collapse and browning of the anther cones, and a protrusion of the stigma from the anther cone of mature flowers [23]. Therefore, a role of JA in male gametophyte development in tomato is suggested, especially in controlling anther senescence and dehiscence.

Dehiscence is typical for maturing stamen [25,26] and represents a coordinated process occurring in specialized cells of the anther that determine the site of anther opening (for review see [27]). This multistage process involves localized cellular differentiation and degeneration to facilitate complete anther opening and pollen release [26]. In tobacco, another *Solanaceae* species, the final events of dehiscence, such as degeneration of the stomium cells and dehydration are affected by ethylene (ET) [27,28]. Treatment of nearly mature anthers with ET accelerated dehiscence, whereas an ET-perception inhibitor retarded dehiscence, and ET-insensitive plants exhibited a loss of anther dehiscence synchrony with flower opening [28]. In tomato, overexpression of *SlERF.B3* encoding a dominant repressor variant of the tomato ET-response factor (ERF), led to a constitutive ET response and sensitivity, which was accompanied by the protrusion of stigma from the stamen cone [29]. These data suggest a scenario where ET acts in stamen development of *Solanaceae* species by regulating stamen dehiscence and pollen release. Whether ET biosynthesis and function might be changed due to JA insensitivity in *jai1-1* is an open question and is addressed by this work.

To get insights into the putative role of jasmonates and the mechanisms involved in stamen development, we performed comparative analyses of stamen development in tomato wild type and *jai1-1* mutant plants in terms of profiling of jasmonates as well as transcript and metabolite profiling. We show that JA levels increased transiently peaking at a middle-bud stage in wild type stamens, whereas they were constantly low in *jai1-1* stamens. *Jai1-1* stamens are characterized by an enhanced expression of ET-regulated genes, higher levels of desiccation-related metabolites and a premature rise of the ET precursor 1-aminocyclopropane-1-carboxylic acid (ACC) starting at the developmental stage of highest JA level in wild type. To further prove these results, application of JA to transgenic plants, which are unable to synthesize JA, and a cross of *jai1-1* with an ET-insensitive mutant (*Never Ripe*, *NR*) were performed. The data suggest a role of JA as inhibitor of a premature function of ET, which itself regulates anther dehiscence and pollen release.

## Results

### Wild type stamens at stage 3 contain highest level of JA and JA-Ile

**Figure 1:**
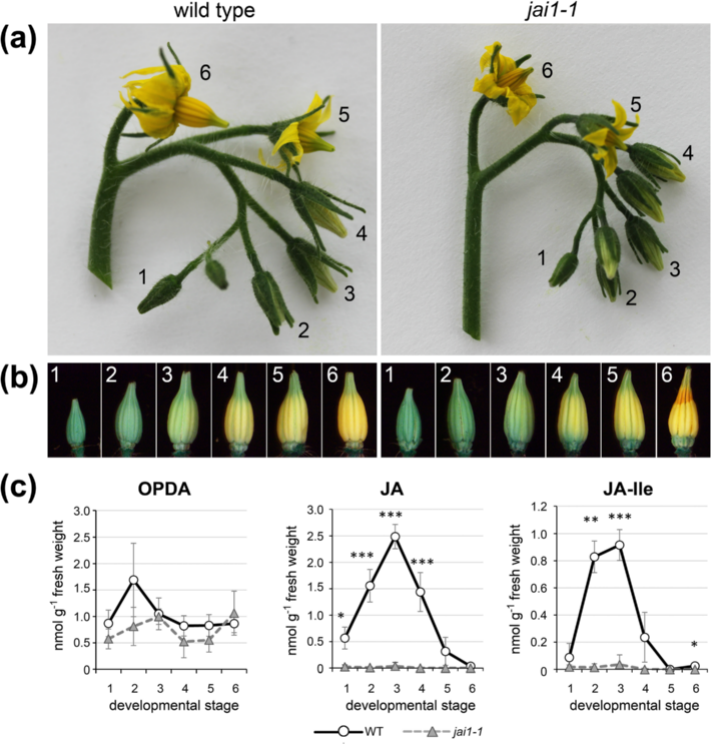
Classification of stages and levels of jasmonates in developing stamens of wild type and *jai1-1*. **(a)** Inflorescences of wild type and *jai1-1* showing the six developmental stages defined for this study. **(b)** Developmental stages of dissected stamen cones of wild type and *jai1-1*. **(c)** Jasmonate levels in developing stamens. Stamens of the respective stages were extracted and contents of OPDA, JA, and JA-Ile were determined. Mean values ± SD are shown. Data of the same developmental stage were compared between wild type and *jai1-1* by Student’s t-test (*P ≤0.05, **P ≤0.01, ***P ≤0.001, n ≥3). JA, jasmonic acid; JA-Ile, JA-isoleucine; OPDA, cis-12-oxo-phytodienoic acid; SD, standard deviation.

Senescence of anther cones is a prominent phenotypic feature of *jai1-1* flowers [23]. To get insights into the processes leading to this stamen phenotype, flower development of *jai1-1* plants and the corresponding wild type was analyzed. Timing of flower development in wild type (cv. MicroTom) and *jai1-1* mutant plants was highly similar. After five weeks of cultivation, the first open flower appeared at the oldest inflorescence and younger flower buds of different stages were visible simultaneously (Figure 1a). The flower bud stages were classified in developmental stages using parameters such as bud size, opening of the sepals and color of the petals. The youngest stage (stage 1 in Figure 1a) represented a small bud completely enclosed by sepals, whereas the oldest stage (stage 6 in Figure 1a) represented the open flower showing bright yellow petals. The stamen cone of *jai1-1* mutant open flowers frequently showed a dehiscent and senescent tip (Figure 1b, stage 6), whereas stamens of other developmental stages did not show obvious phenotypic differences to wild type stamens.

To elucidate the occurrence of jasmonates in male reproductive organs, stamens were collected from each developmental stage of wild type and *jai1-1* plants and the levels of OPDA, JA and JA-Ile were determined (Figure 1c). The OPDA content was similar in all stages and did not differ significantly between wild type and *jai1-1*. This contrasts to the levels of JA and JA-Ile, which both exhibited a significant maximum in wild type stamens at stage 3 and dropped down to levels below the detection limit in stamens of open flowers (Figure 1c). Most importantly, the levels of JA and JA-Ile were almost below the detection limit in *jai1-1* stamens of all developmental stages. Thus, in addition to the JA insensitivity, stamens of *jai1-1* plants are characterized by an absence of active jasmonates possibly caused by the missing positive feed-back in JA biosynthesis and JA-induced gene expression known for developmental processes [1]. As a result, the JA insensitivity might lead to severe alterations in transcript and metabolite accumulations in stamens of *jai1-1* in comparison to wild type.

### Jasmonate-insensitivity alters the transcriptome in stamens

**Figure 2:**
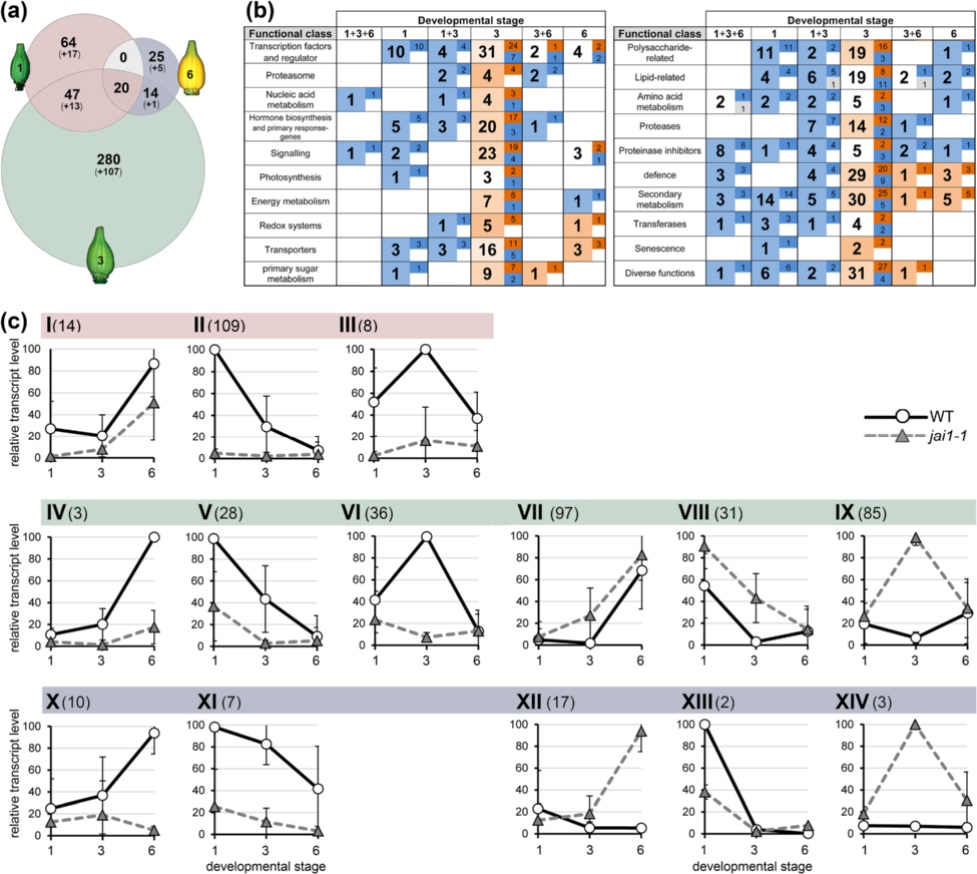
Comparative analysis of transcript accumulation in stamens of wild type and *jai1-1*. Total RNA isolated from three developmental stages of stamens of wild type and *jai1-1* was subjected to transcript profiling using the Agilent-Tomato 44 K-full genome chip. **(a)** Venn diagram showing the number of significantly regulated genes (*P* ≤0.01, n = 3). Numbers in parentheses are related to genes with unknown functions. Note that the highest number of differentially regulated genes was found in stage 3. **(b)** Classification of differentially expressed genes according to functional classes. The bold numbers indicate how many genes in total were differentially regulated in the respective developmental stage, whereas the regular numbers show how many of them exhibited increased transcript levels in wild type (dark red) or *jai1-1* (dark blue). Light red and light blue colors mark the overall tendency of differential transcript accumulation in each developmental stage/functional class (red higher in wild type, blue higher in *jai1-1*). **(c)** Grouping of max-normalized differentially regulated genes according to their kinetics during development. The mean (± SD) of all genes belonging to the respective group is shown, for details of differentially expressed genes within the groups see Figure S1 in Additional file 2. Fourteen groups were generated by application of the following criteria: stage-specificity according to the Venn diagram (I-III: 1, 1 + 3, 1 + 3 + 6 (red), IV-IX: 3 (green), X-XIV: 3 + 6, 6 (blue)), higher expression levels in at least one developmental stage of wild type (I-VI, X-XI) or *jai1-1* (VII-IX, XII-XIV), and kinetics of transcript levels (I, IV, VII, XII: increasing during development, II, V, VIII, XI, XIII: decreasing during development, III, VI, IX, XIV: peaking at stage 3). The Arabic numbers show the number of differentially regulated genes in each group.

To elucidate the role of jasmonates in stamen development, transcript profiling was conducted for stamens of developmental stages characterized by maximum differences in JA/JA-Ile contents. RNA isolated from dissected stamens of stages 1, 3 and 6 of both genotypes was used for hybridization of Agilent Tomato Arrays. To find JA-regulated genes, the Agilent data were quantile-normalized and analyzed using the ArrayStar software as described in the Methods section. The comparison between stamens of wild type and *jai1-1* in each developmental stage revealed 450 genes to be differentially expressed in stamens of both genotypes (Figure 2 and Table S1 in Additional file 1). Most of the identified genes showed stage-specific differential expression. Only a minor part of genes appeared to be differentially expressed in two or three developmental stages. Moreover, a strong correlation between the number of differentially regulated genes and the JA/JA-Ile levels was found. The highest number of regulated genes was detected in stage 3 showing the highest JA/JA-Ile levels in wild type (Figure 2c). It turned out that most of the differentially expressed genes occurring in sections 1, 1 + 3, and 1 + 3 + 6 of the Venn diagram exhibited lower expression levels in *jai1-1* (blue labelled numbers in Figure 2b) pointing to a positive regulatory role of jasmonates in early stamen development. This contrasts to the genes occurring exclusively in stage 3 and 6, in which most of them exhibited higher expression levels in *jai1-1* than in wild type (WT) stamens (red labelled numbers in Figure 2b). Here, a predominantly negative regulatory role of jasmonates in the later stages of stamen development is suggested.

The differentially expressed genes were assigned to 14 groups according to their transcript levels in WT and *jai1-1* and to their kinetics over the three stages analyzed (Figure 2c and Figure S1 in Additional file 2). The majority of genes were classified into groups II, V and VIII showing decreasing transcript levels during stamen development, and into groups VI and IX showing transient increase or decrease at stage 3. Note that especially for groups II and V, the JA responsiveness was stronger in the stage before reaching the maximum of jasmonate levels. Genes in groups VI and IX have opposite profiles of transcript accumulations between WT and *jai1-1* peaking at stage 3, which shows the maximum JA/JA-Ile levels in WT stamens (Figure 1c). Among the positively JA-regulated genes were well-described JA-responsive genes, such as *JAZ1* (SGN-U579837), *JAZ3* (SGN-U564449) and *JAZ8* (SGN-U576446) [12]. Transcript accumulation of these genes was validated by quantitative RT-PCR using RNA from all defined developmental stages of stamen of an independent experiment (Figure S2 in Additional file 2). Transcript levels of all three genes exhibited a transient maximum at stage 3 correlating with the maximum JA/JA-Ile levels. The gene encoding the JA biosynthetic enzyme AOC, however, did not show a differential regulation between WT and *jai1-1* (Figure S2 in Additional file 2) pointing to a preferentially developmental regulation of its expression. This is reflected by the level of OPDA, which was not significantly different between WT and *jai1-1* (Figure 1c).

All identified differentially expressed genes could be assigned to 20 functional classes (Figure 2b). The highest numbers of differentially expressed genes occurred in the classes ‘transcription factors and regulators’ (51), ‘signaling’ (29), ‘defense’ (40) and ‘secondary metabolism’ (58). This coincides with a putative role of jasmonates due to the well-described functions of jasmonate-induced genes [1]. Additionally, genes encoding lipid transfer proteins, peptide transporters and UDP glucosyltransferases were among the positively JA-regulated genes showing a diminished expression in early stamens (stage 1) of *jai1-1* in comparison to WT (Table S1 in Additional file 1). Their putative gene products might be involved in pollen nutrition.

**Figure 3:**
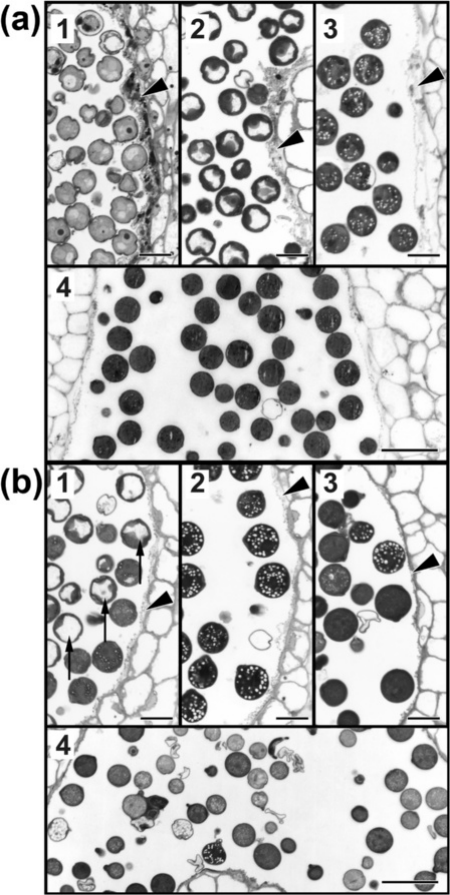
Pollen development in wild type and *jai1-1*. Semi-thin cross-sections of anthers of wild type **(a)** and *jai1-1* **(b)** stained with toluidine blue. The developmental stages are indicated by numbers. Note the high number of aborted pollen grains in *jai1-1* (b 4). Pollen of *jai1-1* appear premature in development as shown by typical features, such as occurrence of the big central vacuole (arrows in b1), presence of starch grains (b1 – b3) and the glassy state (b3) appearing first in wild type from stage 4 onwards. Additionally, premature degeneration of tapetum (arrow heads) is visible in anthers of *jai1-1*. Bars represent 20 μm in (a1-3) and (b1-3), and 50 μm in (a4) and (b4).

**Figure 4:**
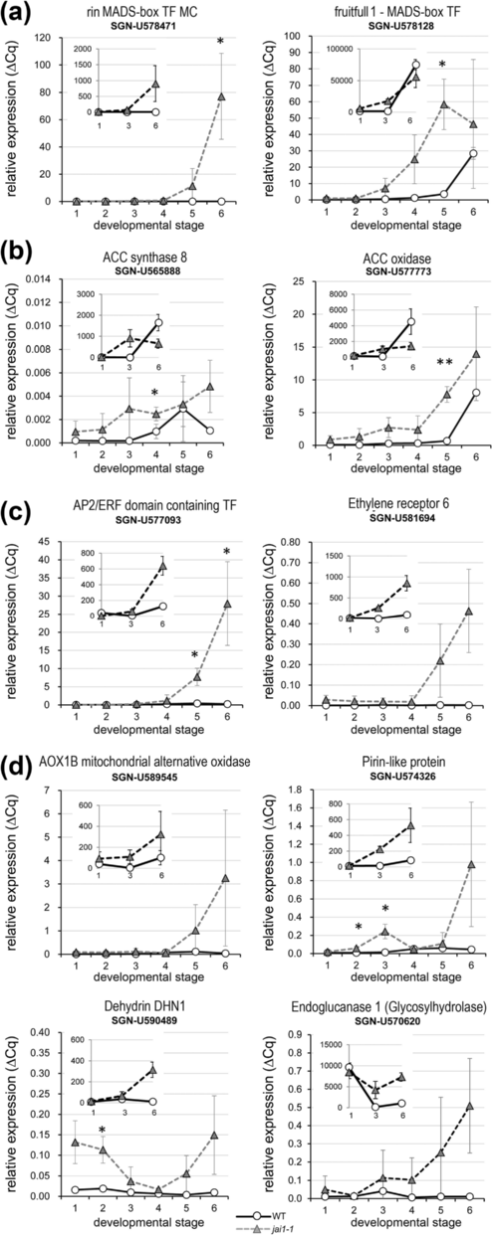
Transcript accumulation pattern of ethylene-related genes in developing stamens of wild type and *jai1-1*. Relative transcript levels of genes encoding ET-related TFs acting as ‘master regulators’ **(a)**, ET biosynthetic enzymes **(b)**, proteins involved in ET-signaling **(c)**, and of ET-response genes **(d)**. All transcript levels were determined by RT-qPCR and set in relation to *SlTIP41*. The inset in each diagram visualizes the signal intensity obtained from microarray analysis. Mean values ± SD are shown. Data of the same developmental stage were compared between wild type and *jai1-1* by Student’s t-test (*P ≤0.05, **P ≤0.01, n = 3). ET, ethylene; SD, standard deviation.

Therefore, pollen morphology and development was analyzed using cross sections of chemically fixed and embedded stamens (Figure 3). In the WT stamen at stage 1, late unicellular microspores were detected showing small vacuoles (Figure 3a, 1). Pollen at bud stage 2 was in the bicellular stage and exhibited a large central vacuole (Figure 3a, 2). Simultaneously, the tapetum started to degrade. At stage 3, all pollen grains showed a high number of starch grains, and the vacuoles had disappeared (Figure 3a, 3). During the following stages, starch appeared to be degraded and the cytoplasm reached a glassy state (Figure 3a, 4). In *jai1-1* stamens, however, different developmental pollen stages were detectable simultaneously and the development of microspores and pollen occurred faster than in WT stamens. The morphological features described for the WT appeared at least one stage earlier. Central vacuoles and starch grains appeared in pollen of stage 1 and 2, respectively, and the cytoplasm was in the glassy state already at stage 3 (Figure 3b, 1-3). Most importantly, the tapetum was not detectable in stage 1 accompanied by a high number of aborted pollen at the final stage of the development (Figure 3b, 4). The abnormal morphology of pollen of *jai1-1* supports the assumption drawn from transcript data that jasmonates are important for the timing of pollen development and might affect the nutrient supply of developing pollen.

Searching the differentially expressed genes for candidates that might have regulatory functions in late stamen development, genes related to ET-dependent processes stood out (Table S1 in Additional file 1). Among these, genes encoding the following classes of proteins were identified: ET-related TFs acting as ‘master regulators’ such as RIN-MADS TF (SNG-U578471) and MADS-Fruitfull TF1 (SGN-U578128), enzymes involved in ET biosynthesis such as 1-aminocyclopropane-1-carboxylic acid (ACC) synthase 8 (ACS8, SGN-U565888) and ACC oxidase-like (SGN-U577773), proteins involved in ET signaling such as AP2/ERF domain containing TF (SGN-U577093) and ET receptor 6 (SGN-U581694) as well as ET-induced proteins such as mitochondrial alternative oxidase AOX1B (SGN-U5589545), pirin-like protein (SGN-U574326), dehydrin DHN1 (SGN-U590489) and endoglucanase 1 (SGN-U570620).

For a validation of these selected candidates, we used a new set of plants and all defined developmental stages of stamen to determine their relative transcript levels by quantitative RT-PCR (Figure 4). Transcript levels of all selected genes were at the detection limit at early developmental stages of WT stamens and started to rise earliest in stage 5 (SGN-U578128) or stage 6 (SGN-U592775). In stamens of *jai1-1*, however, these transcripts were detected at earlier stages and to higher levels. These data suggest that the absence of JA/JA-Ile and/or jasmonate signaling in *jai1-1* stamen might lead to an early or premature ET biosynthesis and action and subsequently might contribute to the early senescence and dehiscence observed in stamen of *jai1-1*.

### Jasmonate-insensitivity alters the steady state levels of soluble metabolites in stamens

**Figure 5:**
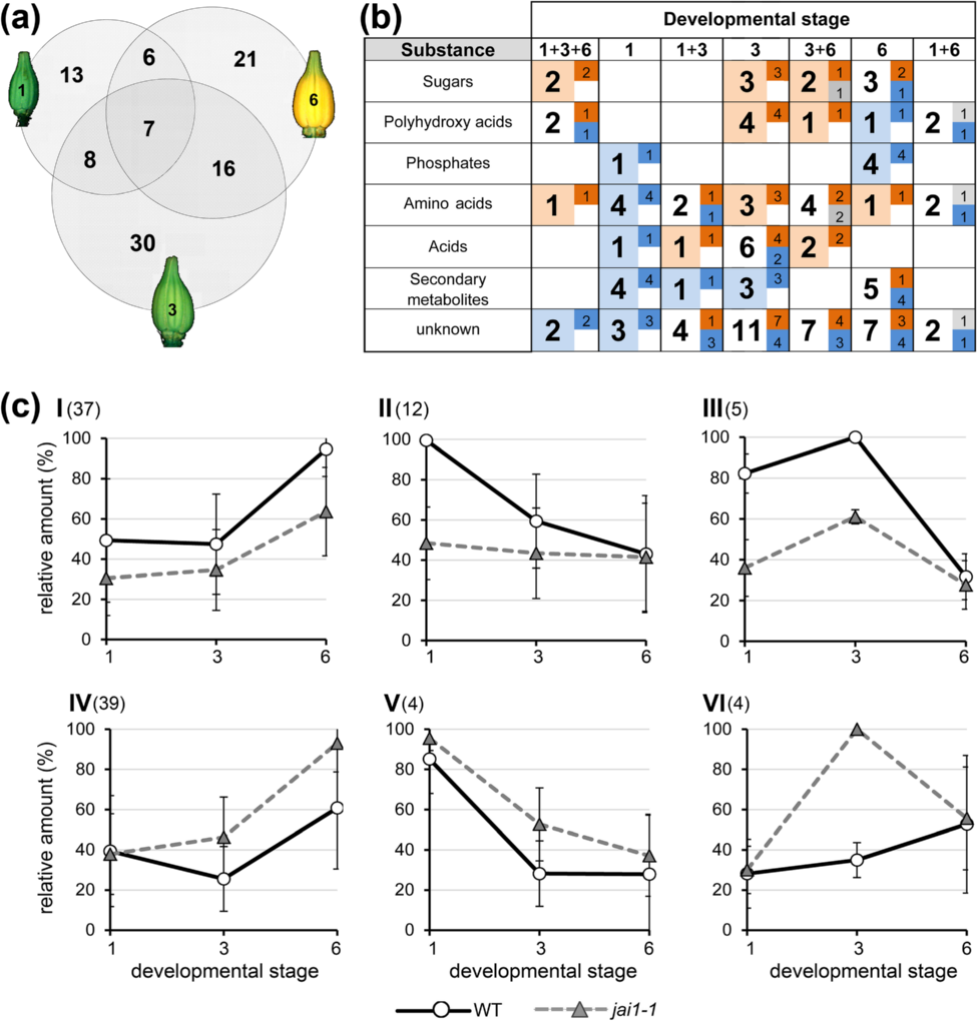
Comparative steady-state analysis of soluble metabolites in stamens of wild type and *jai1-1*. Soluble metabolites were extracted from stamens of three developmental stages and analyzed by non-targeted metabolite profiling. Levels of metabolites were compared between wild type and *jai1-1* in each developmental stage. **(a)** Venn diagram showing the number of metabolites accumulating in significantly different amounts in wild type and *jai1-1* stamens (*P* ≤0.05, n = 6). Note that the highest number of differentially occurring metabolites was found in stage 3. **(b)** Classification of differentially occurring metabolites according to their chemical nature. Numbers in bold indicate how many metabolites occurred in different amounts in total in the respective developmental stage, whereas the small numbers show how many metabolites of them exhibited increased levels in wild type (dark red) or *jai1-1* (dark blue). Light red and light blue colors mark the overall tendency of differential accumulation in each developmental stage/substance class (red higher in wild type, blue higher in *jai1-1*). **(c)** Grouping of max-normalized metabolites according to their kinetics during development. The mean (± SD) of all metabolites belonging to the respective group is shown, for details of differentially occurring metabolites see Figure S3 in Additional file 2. The following criteria were applied for building groups: higher accumulation according to the Venn diagram in at least one developmental stage of wild type (I-III) or *jai1-1* (IV-VI), and kinetics of metabolite levels (I, IV: increasing during development; II, V: decreasing during development; III, VI: peaking at stage 3). The Arabic numbers show the number of metabolites in each group. Note that most metabolites show an accumulation during stamen development. SD, standard deviation.

Metabolite profiling was performed using stamens of stage 1, 3 and 6, which were collected and subjected to non-targeted metabolite profiling. By analyzing polar compounds using gas chromatography-mass spectroscopy (GC-MS), we could detect about 100 compounds showing significantly different levels in stamens of WT and *jai1-1* in at least one developmental stage (Table S2 in Additional file 1). From these, around two thirds could be assigned to known substance classes (Figure 5). Most of the differential compounds were detectable in stage 3 (Figure 5a), which had the highest JA levels in wild type stamens. Comparing the levels of the identified compounds, it became obvious that in stage 1 most of them exhibited a lower content in *jai1-1* than in WT, whereas differentially occurring compounds in stamens of stages 3 and 6 showed higher levels in *jai1-1* (Figure 5b).

Six different groups of metabolites were defined according to the different levels in WT and *jai1-1* stamens and to their kinetics during development from stage 1 to stage 6 (Figure 5c and Figure S3 in Additional file 2). Only a few metabolites exhibited a decrease during development or a transient increase peaking at stage 3, whereas nearly 80% of the identified metabolites belonged to groups I and IV showing an accumulation during development. Among them, about 60% of metabolites showed higher levels in *jai1-1* than in WT stamens (group IV in Figure 5c). In group IV, mainly sugars, sugar acids and amino acids were found. As exemplified for selected monosaccharides (glucose, fructose), di- and trisaccharides (sucrose, raffinose, trehalose, 1-kestose), sugar alcohols (*myo*-inositol, galactinol), and amino acids (proline, glycine, valine, methionine), these metabolites showed low levels in WT stamens at stage 1, followed by a slight change at stage 3 and a moderate increase at stage 6 (Figure S4 in Additional file 2). In *jai1-1* stamens, however, some of them already showed higher levels at stage 1, many of them showed high levels at stage 3, and all of them increased drastically at stage 6 resulting in contents up to six-fold higher than those in WT stamens at this stage (Figure S4 in Additional file 2). Photometric measurements of glucose, fructose and sucrose performed for all six defined developmental stages confirmed the data obtained by metabolite profiling and revealed a continuous accumulation of these sugars in stamens of *jai1-1* resulting in significantly higher levels than in WT stamen (in Figure S5 in Additional file 2). This contrasts to sugar phosphates, which accumulated over development in WT stamens, but exhibited lower levels in *jai1-1* stamens (Table S2 in Additional file 1).

**Figure 6:**
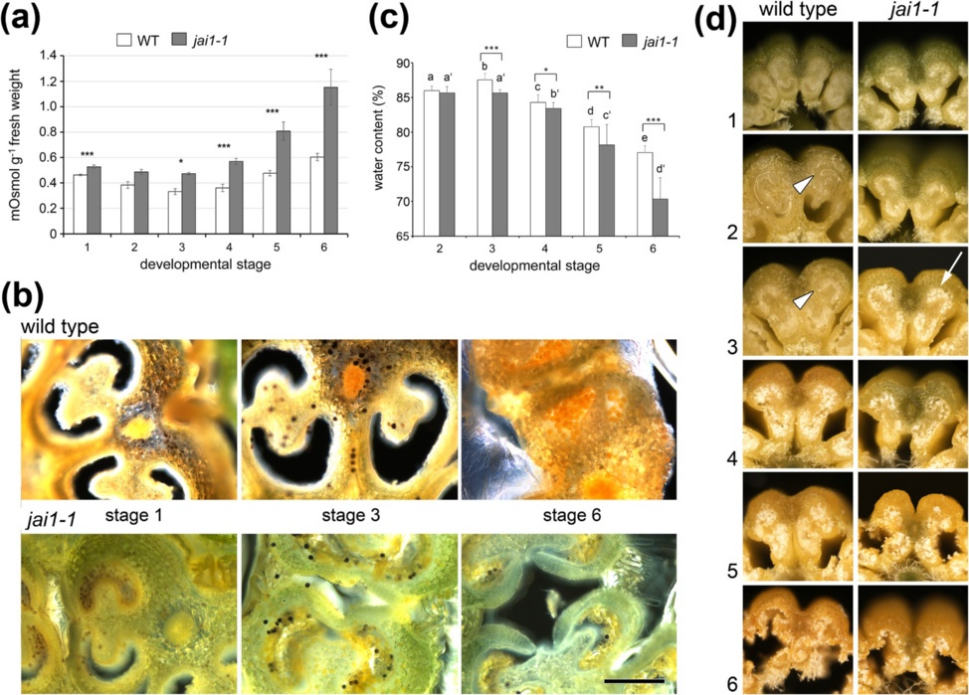
Premature dehiscence in stamens of *jai1-1*. **(a)** Osmolality in stamens of wild type and *jai1-1* plants. **(b)** Occurrence of starch in anther tissues of wild type and *jai1-1* plants. Sections of fresh stamens were stained with iodine potassium iodide resulting in a black and brownish coloration of amylose and amylopectin, respectively. Note the strong staining in stamen of wild type plants showing a high number of starch grains and high levels of amylopectin, whereas stamen of *jai1-1* show only a few starch grains and almost no amylopectin. Bar represents 100 μm for all micrographs. **(c)** Relative water content in stamens of wild type and *jai1-1*. **(d)** Cross-sections of fresh stamens of the six developmental stages in wild type and *jai1-1*. Note the appearance of fluids in the locule of wild type stamen at stage 2 and 3 (arrow heads) being absent in *jai1-1*. In contrast, *jai1-1* pollen appear whitish (arrows) at early developmental stages pointing to premature pollen desiccation and accumulation of desiccation-related substances. Mean values + SD of stamens of the developmental stages indicated are shown in **(a)** and **(c)**. *P ≤0.05, ***P ≤0.001 according to Student’s t-test (n ≥ 8). SD, standard deviation.

Taken together, the enhanced content of sugars as well as amino acids known as protective compounds in response to osmotic stress [30] points to increasing water loss attributing to the premature dehiscence in *jai1-1* stamens. Interestingly, the higher levels of osmotically active compounds coincided with a higher osmolality of the cellular fluid in *jai1-1* stamens (Figure 6a). In all developmental stages, *jai1-1* stamens exhibited a significantly increased osmolality, reaching at stage 6 about twice the levels measured in WT stamens. This was accompanied by a diminished accumulation of starch in stamens of *jai1-1* in comparison to WT stamens as visualized by staining of fresh sections (Figure 6b).

### Jasmonate-insensitivity alters ethylene production and function in stamens

**Figure 7:**
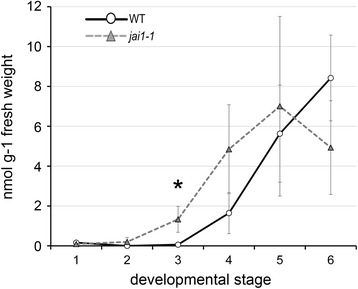
Levels of ACC in developing stamens of wild type and *jai1-1*. Stamens of the respective stages were extracted and content of ACC was determined. Mean values ± SD are shown. Data of the same developmental stage were compared between wild type and *jai1-1* by Student’s t-test (* *P* ≤0.05, n = 4). ACC, 1-aminocyclopropane-1-carboxylic acid; SD, standard deviation.

The transcriptome and metabolite data indicate an early rise in ET levels in stamen of *jai1-1*. Therefore, the content of its precursor ACC was determined in stamens of WT and *jai1-1* (Figure 7). Indeed, the rise in ACC occurred in *jai1-1* at least one stage earlier than in WT stamens and is highly correlated to the expression profiles of ET biosynthesis and ET response genes (see Figure 4). Altogether, this points to a premature ET biosynthesis and signaling in *jai1-1* stamens that in WT stamen might be down-regulated by jasmonates.

**Figure 8:**
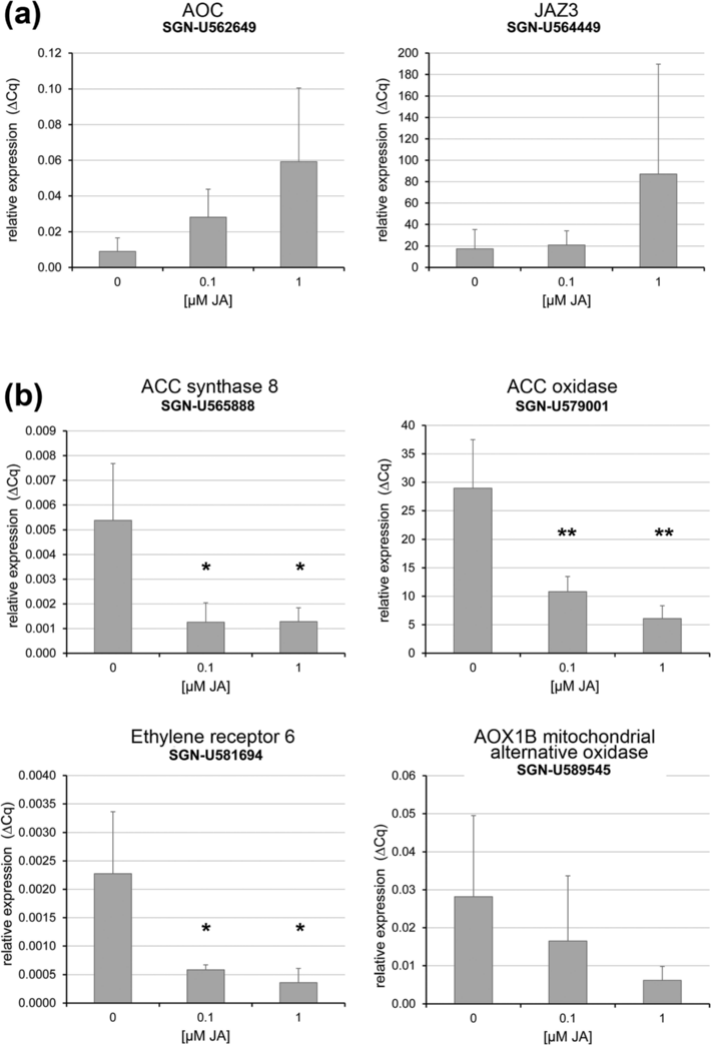
Transcript accumulation of selected JA-responsive and ET-related genes in stamens of JA-treated flowers buds. Flower buds in stage 2 were treated three times in an interval of 20 hours by spraying with JA solution in the concentration indicated. Stamens were harvested from flower buds at stage 5 (five hours after the last treatment). **(a)** Relative transcript levels of genes encoding early JA-induced genes in stamen of wild type plants. **(b)** Relative transcript levels of genes encoding ET biosynthetic enzymes, proteins involved in ET-signaling, and of ET-response genes in stamen of *AOC-RNAi* plants. All transcript levels were determined by RT-qPCR and set in relation to *SlTIP41*. Mean values + SD are shown. Data of JA treated stamen were compared with stamen of control treatment (0 μM JA) by Student’s t-test (*P ≤0.05, **P ≤0.01, n = 4). ET, ethylene; JA, jasmonic acid; SD, standard deviation.

To verify this hypothesis, we applied JA to developing flower buds of tomato. Since WT stamen already contain high levels of JA/JA-Ile (Figure 1c), which might be sufficient for down-regulation of ET-related genes, transgenic Micro-Tom plants expressing an *SlAOC-RNAi* construct under control of the *35S* promoter were used [31]. These plants are characterized by diminished JA levels and exhibit a *jai1-1*-like phenotype. Repeatedly performed application of JA to WT flower buds starting at stage 2 resulted in enhanced transcript levels of JA-regulated genes in stamen of stage 5 (Figure 8a). In stamen of *SlAOC-RNAi*, however, a significant down-regulation of selected ET-related genes, such as those encoding ACS8, ACO, ERF-6 and AOX1B, was detected (Figure 8b). This down-regulation of ET related transcripts points to a negative regulatory role of JA in late stamen development.

**Figure 9:**
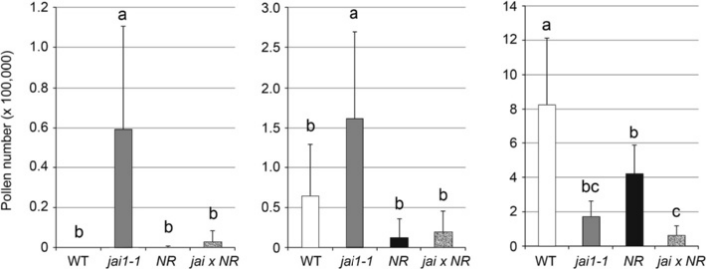
Pollen release from anthers of wild type, *jai1-1* and mutants affected in ET-signaling. Dissected anthers of the developmental stage 4s **(a)**, 5 **(b)** and 6 **(c)** of wild type (WT), *jai1-1*, *never ripe* (*NR*), *epinastic* (*epi*) and the double mutant *jai1-1 NR* were shaken in PBS and released pollen were counted. The overproduction of ET in *epi* resulted in similar premature pollen release at stage 4 as detectable for *jai1-1*, whereas pollen release in *NR* and *jai1-1 NR* is similar to WT. Note that the absolute numbers of released pollen are less in *jai1-1*-based genotypes due to the high number of aborted pollen (see Figure 3). Mean values + SD are shown (n ≥13). Different letters designate statistically different values (ANOVA with Tukey’s HSD test; *P* ≤0.01). ANOVA, analysis of variance; ET, ethylene; HSD, honest significant difference; SD, standard deviation.

To prove the hypothesis that JA insensitivity accompanied by premature ET action leads to early dehiscence, water content in developing stamens was determined (Figure 6c). Wild type stamens showed increasing water content up to stage 3 followed by a slight, but constant loss of water until anther maturation (stage 6). In contrast, *jai1-1* stamens did not show increasing water content in stage 3 and exhibited a much stronger water loss during further development resulting in a maximum difference to WT at stage 6. Cross sections of fresh stamen cones revealed that stamens of WT flower buds of stages 2 and 3 exhibited a high amount of locular fluid filling the space between developing pollen grains (Figure 6d). This fluid was nearly absent in *jai1-1* anthers of the same developmental stage correlating with the differences in water content. Consistently, the premature dehiscence is reflected also by premature pollen release from *jai1-1* stamens tested by shaking anthers followed by counting the released pollen (Figure 9). Only a few pollen were released from WT stamens at stage 5 and the majority of pollen was released in stage 6, whereas pollen release in *jai1-1* started already in stage 4. This is also reflected by the closed stomia of WT stamen at stage 4, whereas they are already opened in *jai1-1* stamen at the same stage (Figure S6a, b in Additional file 2). In summary, the lower water content, the absence of fluid in the locule of developing stamens, and the premature release of pollen in *jai1-1* point to a deregulated function of ET in control of water content and dehiscence of tomato stamens not only in the mature stage, but already during development of the flower bud.

To test this, pollen release in mutants affected in ET perception, such as *Never Ripe* (*NR*, [32]) or showing a constitutive ET response, such as *epinastic* (*epi*, [33]), both in the Micro-Tom background [34], was determined in comparison to WT and *jai1-1* (Figure 9). Consistent with ET function in senescence and dehiscence, *NR* anthers exhibited a delayed pollen release, whereas *epi* anthers showed a premature pollen release. Here, similar to *jai1-1*, released pollen were already detectable in *epi* stamen of stage 4, whereas almost no pollen were released from stamen of WT and *NR* in this stage. This leads to the conclusion that jasmonate-insensitivity in *jai1-1* mimics the constitutive ET response in the *epi* mutant.

As genetic proof, *jai1-1* and *NR* were crossed and stamens of the double mutant were analyzed regarding pollen development and release. As visible in semi-thin cross sections of *jai1-1 NR* anthers, pollen development at early stages was similar to that of the *jai1-1* single mutant (Figure S7 in Additional file 2). The development of microspores and pollen occurred faster than in WT and *NR* stamens and the tapetum was degraded in stage 1. From these data we conclude that at early stages, which are not related to ET function, *jai1-1* dominates *NR* and introduction of ET insensitivity did not change the *jai1-1* phenotype. However, a contrasting picture appeared at late stages of stamen development. Here, ET insensitivity rescued the *jai1-1* phenotype. Pollen release and closure of stomium of the double mutant at stage 4 is similar to *NR* (Figure 9, Figure S6c, d in Additional file 2). The data obtained by these approaches support the assumption that jasmonates function as inhibitors of premature rise of ET during stamen development in WT tomato.

## Discussion

Jasmonates, known as stress hormones, are also involved in the regulation of various developmental processes of plants. Jasmonates are crucial for plant fertility, since JA deficiency or insensitivity in *A. thaliana* results in male sterility characterized by delayed anther dehiscence, insufficient filament elongation and production of unviable pollen [20,35]. In contrast, JA insensitivity in tomato leads to a female sterile phenotype, although the development of stamen and pollen is also affected [23]. Therefore, *jai1-1* stamens provide a useful tool for identifying JA and COI1-dependent processes that operate in male reproductive tissues of tomato. Comparing *jai1-1* and WT stamens in terms of gene expression and metabolite accumulation, this study revealed that jasmonates regulate pollen nutrition and thereby pollen development, but most obviously they are an important regulator of the proper timing of stamen dehiscence via a temporal repression of ET biosynthesis and action.

In developing stamens of WT plants, JA and JA-Ile levels exhibited a maximum at an intermediate developmental stage (Figure 1), which was defined as buds of maximal size with opened sepals, but white petals. At this stage, stamens undergo a switch between the two developmental phases characterized as the histodifferentiation program (phase l of stamen development) and cell degeneration and dehiscence program (phase II of stamen development) [36]. Up to flower bud stage 3, developing pollen grains finished pollen mitosis, took up water and sugars from locular fluid and started to accumulate starch (Figure 3). The maximum jasmonate level coincided with the number of differentially accumulating transcripts in WT and *jai1-1* stamens. In stages before reaching the JA maximum, that is, during phase I of stamen development, most of the differentially regulated genes exhibited higher transcript accumulation in WT than in *jai1-1* stamens (Figure 2), indicating a positive regulatory role of jasmonates in phase I. Additionally, gene expression analysis revealed that positive regulation by JA appeared to be stronger in stages before reaching jasmonate maximum. Interestingly, this group contains genes, whose putative gene products might be responsible for pollen nutrition and water homeostasis in pollen and anther tissue. These alterations in expression pattern of *jai1-1* stamens were accompanied by reduced metabolite levels, but also by a premature degradation of the tapetum, a high number of aborted pollen and the absence of locular fluid (Figures 3, 5 and 6).

Formation of viable pollen within the locules of the anther is dependent on nutritive contributions from the surrounding sporophytic tissue, and pollen development relies on the nutritive support of neighboring tapetal cells [21,37]. Developing microspores obtain their carbohydrate supply from the tapetum [38] and from degradation of callose surrounding the microspore tetrads [39]. During maturation, pollen grains receive their sugars directly from the locular fluid, since at the later stages of anther development the tapetum no longer exists [40]. The locular fluid contains large amounts of soluble compounds as a result of polysaccharide degradation [41]. However, premature death of tapetal cells results usually in the disruption of the nutrient supply to the microgametophytes, leading to their death [42,43]. Therefore, the premature degradation of the tapetum and the absence of the locular fluid in *jai1-1* stamens may contribute to an inefficient pollen nutrition followed by abortion of microspores as well as by an accelerated and inefficient maturation of the remaining pollen. The diminished formation of vacuoles in pollen of *jai1-1* plants (see Figure 3) might point to such an insufficient water and sugar uptake since both processes are assumed to be linked [41].

A different scenario was obtained upon transcript and metabolite profiling of stamens in phase II of development. In stages after the JA maximum, most of the differentially regulated genes appeared to be expressed at higher levels in *jai1-1* than in WT stamen implicating a negative regulatory role of JA. Most prominently, transcripts of genes involved in ET biosynthesis (ACC synthase, ACC oxidase), ET signaling (transcription factors such as RIN, FRUITFULL, AP2/ERF, and receptors such as ETR6) and ET-response genes (AOX1B, Pirin-like, DEHYDRIN) exhibited higher levels in *jai1-1*. This was accompanied by a higher content in the ET precursor ACC, whose levels increased at least one stage earlier in *jai1-1* than in WT stamens. Accordingly, some of the genes were down-regulated by application of JA to flower buds of a *SlAOC-RNAi* line (Figure 9) supporting the hypothesis that jasmonates control biosynthesis and function of ET in a negative way.

Among the ET response genes were genes encoding polysaccharide degrading enzymes, such as endoglucanase 1 (Figure 4). This is in line with the fact that hydrolytic enzymes and proteins linked to cell wall loosening are thought to be involved in anther dehiscence [27]. Along with that, osmotic active metabolites, such as proline, non-reducing sugars, such as sucrose, trehalose and 1-kestose, as well as protective proteins, such as dehydrin, were found to be accumulating in higher amounts in *jai1-1* than in wild type stamen (Figures S4 and S5 in Additional file 2). The higher hexose levels in *jai1-1* stamens might arise from an insufficient conversion of starch from sugars. Indeed, less starch was found in the anther tissues of *jai1-1* stamens than in anthers of WT stamens, where a maximum of starch accumulation was detected in stage 3 and possibly resulted in a chemical formation of water (Figure 6). In turn, higher glucose and fructose contents in the *jai1-1* might also function as osmoprotectants to compensate the loss of water released upon starch biosynthesis. Together, this could result in an increased dehydration within specific cell types and regions of the anther (Bonner and Dickinson, 1990). Certainly, the stomia of *jai1-1* stamens exhibited a premature rupture (Figure S7 in Additional file 2) contributing to the early pollen release.

The enhanced expression of ET-related genes and the early accumulation of ACC in *jai1-1* stamens suggest that a premature rise and action of ET occur in stamens of *jai1-1* and leads to dehiscence and pollen release. This is consistent with the delayed and premature pollen release observed in stamen of the tomato mutants *NR* and *epi*, respectively (Figure 9), thereby supporting the role of ET in dehiscence and pollen release (Scott *et al*., [26]). Most importantly, the genetic proof by creating the double mutant *jai1-1 NR* showed unequivocally that ET insensitivity led to a rescue of the phenotype of *jai1-1* stamen at late developmental stages (Figure 9), but not at early stages (Figure S7 in Additional file 2). This supports the hypothesis that jasmonates have a positive regulatory role at phase I of stamen development, which is not related to ET, whereas jasmonates are necessary to prevent the early rise and action of ET in phase II regulating senescence and dehiscence.

ET and JA are found to be coordinately (cooperatively or antagonistically) regulated or exhibit opposite effects on many plant responses [1,44,45]. A synergistic cross-talk between JA and ET is known to occur preferentially for the response to necrotrophic pathogens [46], whereas they act antagonistically in regulation of the expression of wound-responsive genes [47,48] and metabolite biosynthetic genes [49]. Furthermore, jasmonates repress apical hook formation [50], while ET has the opposite effect [51]. In *Arabidopsis*, JA and ET act in parallel to regulate timing of floral organ abscission [52]. Depending on developmental stages and physiological responses, JA affects ET signaling downstream of the ET receptors, since reduced JA levels might cause ET sensitivity in originally ET insensitive *ein2* mutants [53]. Recently, a model for the molecular mechanism for the antagonism between JA and ET signaling in the apical hook formation in *A. thaliana* was presented [44,54]: on the one hand, MYC2, a prominent member of the MYC-transcription factor-family activated by JA, can physically interact with the ET-activated transcription factor ETHYLENE INSENSITIVE3 (EIN3) to directly inhibit its transcriptional activity. On the other hand, MYC2 positively regulates expression of EIN3 BINDING F-BOX PROTEIN1 (EBF1) by directly binding to its promoter leading to an increase in EBF1-promoted EIN3 degradation. However, applying strong statistical criteria neither *MYC2* nor *EBF1* were found among the differentially regulated genes in our dataset from tomato stamen.

## Conclusions

**Figure 10:**
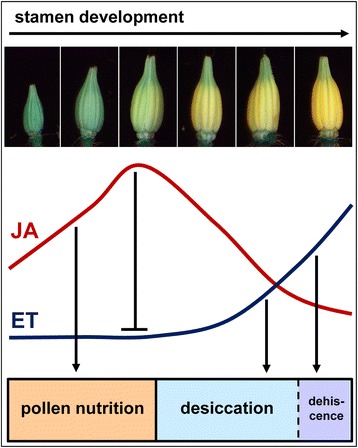
Schematic model of the role of jasmonates in stamen development of tomato. Jasmonate levels increase transiently during stamen development. In the first phase, increasing JA levels promote pollen nutrition and development. In the second phase of stamen development, jasmonates function as inhibitor of a premature rise of ethylene, which itself positively regulates anther dehiscence and pollen release.

The characteristic phenotypic feature of *jai1-1* flowers is a premature dehiscence and senescence of stamens visible by browning of the cone tip [23] leading to the hypothesis that function of the senescence-promoting hormone ET might be deregulated in this mutant. Obtained by global transcript and metabolite profiling, the presented data reveal a new scenario for the function of jasmonates and ET in the stamen development of tomato (Figure 10). During early stamen development jasmonates seem to promote processes related to pollen nutrition and water supply. In the later developmental stages, JA insensitivity in the *jai1-1* mutant leads to a premature expression of some of the well-known master regulators in ET signaling, thereby leading to premature biosynthesis and action of ET.

The premature dehiscence of *jai1-1* stamen is in contrast to the known effects of JA deficiency or insensitivity in *A. thaliana*, where in the corresponding mutants dehiscence of anthers appears to be delayed. In developing flowers of *Arabidopsis* WT plants, the highest JA levels occurred in the mature flower [55], and JA is absolutely required in stamen of flower stage 12 to enable filament elongation in the subsequent developmental stage [17]. This confirms a function of JA as a positive regulator in *Arabidopsis* to trigger the developmental switch that initiates the program for final stamen maturation and dehiscence. In tomato, however, the JA/JA-Ile content already started to decline at a stage before the desiccation process starts. Therefore, it is likely that jasmonates are not directly involved in the regulation of dehiscence of tomato anthers, but fulfill a regulatory role by controlling the dehiscence-promoting hormone ET to prevent a premature occurrence of this process. With this model, a new form of cross-talk between JA and ET in a plant developmental process becomes obvious. The elucidation of regulatory components that mediate this cross-talk will deliver new insights into the regulatory role of JA and ET in the development of tomato stamen.

## Methods

### Plant material, growth conditions, harvest of stamens, and JA treatment

*Solanum lycopersicum* plants cv. MicroTom wild type (WT), *jai1-1* [23], *NR* and *epi* [34]*,* and transgenic plants expressing *35S::AOC-RNAi* [31] were grown in in a controlled growth chamber with 16 hours light (300 μmol photons*m^−2^*s^−1^) and 8 hours dark, both at 28°C and 50% humidity. Homozygous *jai1-1* plants were selected by PCR according to [23]. Phenotypic markers, such as missing anthocyanin production in first leaves, protrusion of stigma and trichome-free fruits were additionally used for selection. *AOC-RNAi* expressing plants were selected by determination of *AOC* transcript levels in leaves one hour after mechanical wounding (see below).

Crosses of *jai1-1* and *NR* were performed by fertilization of *NR* ovaries with *jai1-1* pollen. Seeds of the resulting F1 generation were germinated on 200 μM ACC to select ET-insensitive plants, which do not show the typical triple response. Plants being homozygous for *jai1-1* were subsequently selected by PCR according to [23].

Harvest of stamens was performed using five- to six-week old plants showing the first open flowers. Stamens of defined developmental stages were harvested in a very strict time window of 30 minutes starting at four hours after the onset of the light period, collected on dry ice, transferred to liquid nitrogen and stored at -80°C. Stamens of all inflorescences were harvested for metabolite measurements, whereas those of primary inflorescences were used for transcript profiling. Harvesting time for stamens subjected to phytohormone quantification was expanded to 90 minutes and stamens of primary and secondary inflorescences were used.

Treatment of flower buds with JA was done by spraying 260 μl of an aqueous JA solution (0.1 μM and 1 μM) three times during an interval of 20 hours starting with buds at stage 2. During this time, flowers buds developed to stage 5 and stamen were harvested five hours after the last treatment. Spraying with water served as the control treatment.

### RNA isolation and qRT-PCR

RNA isolation from homogenized material was performed using the RNAmini plant Kit (Qiagen, Hilden, Germany [56]) according to the supplier’s instructions including on-column digestion of DNA for 30 minutes. RNA quality was tested by capillary electrophoresis using the QIAxcel Advanced System (Qiagen). First strand cDNA synthesis of 1 μg RNA was performed in a final volume of 20 μl with M-MLV Reverse Transcriptase, RNase H Minus, Point Mutant (Promega, Fitchburg, WI, USA) according to the supplier’s protocol using oligo(dT)19 primer.

A total of 3 μl of cDNA (diluted 1:20) were mixed with 2 μl 5× EvaGreen QPCR Mix II **(Bio**&Sell, Feucht, Germany [57]), 2 pmol forward primer and 2 pmol reverse primer and dH_2_O (at 10 μl). QPCR primers for candidate genes were designed with the software CloneManager (Sci-Ed Software, [58]) using the corresponding sequences of the tomato genome (sol genomic network, [59]) (for primer sequences see Additional file 2: Table S3). PCR was done using qRT-PCR-System CFX Connect (Bio-Rad, München, Germany [60]) with the following protocol: denaturation (95°C for 15 minutes), amplification (40 cycles of 95°C for 15 seconds and 60°C for 30 seconds) and melting curve (95°C for 10 seconds, 60°C heating up to 95°C with a heating rate of 0.5°C s^−1^). Data were analyzed with CFX Manager Software (Bio-Rad). Relative gene expressions were calculated by the comparative Cq method [61] using *SlTIP41* [62] as the constitutively expressed gene. Each reaction was measured in triplicate.

### GeneChip data analysis

RNA isolated independently from three stamen samples per developmental stage (1, 3 and 6), all from WT and *jai1-1*, was analyzed using Agilent-Tomato 44 K-full genome chips. Synthesis and purification of cDNA; synthesis, labeling, purification, quality control and fragmentation of cRNA; as well as hybridization, washing, and scanning of the chips were done by the service partner (Atlas Biolabs, Berlin, Germany) [63] according to the supplier’s protocols.

Data analysis was performed using ArrayStar [64]. All samples were quantile normalized. To identify genes that were differentially expressed, pairwise comparison between WT and *jai1-1* at the three different developmental stages were done. *P*-values were corrected according to the Benjamini-Hochberg method using the statistical package of ArrayStar. Genes were considered as differentially expressed when adjusted *P*-values were ≤0.01 and fold change ≥8. Sets of genes showing differential expression were obtained for each developmental stage. Gene annotation was done using data from ‘Sol Genomics Network’ and mapping results obtained by MapMan [65] followed by manual check and correction using nucleotide blast [66]. Generation of groups was performed with Excel as described for metabolites. The original Agilent GeneChip data as well as normalized data from this study are publicly available at ArrayExpress database [67] under accession number E-MTAB-2752.

### Determination of OPDA, JA, JA-Ile and ACC

Quantitative analysis of OPDA, JA and JA-Ile was done using 500 mg of homogenized plant material per sample as described [24]. To determine content of ACC, 500 mg of frozen and grinded material was extracted with 10 ml methanol supplied with 50 ng [^2^H_4_]-ACC as internal standard. The homogenate was filtered and placed on a column filled with 3 ml DEAE-Sephadex A25 (GE Healthcare, München, Germany [68]) followed by washing with 3 ml methanol. The flow through was evaporated, dissolved with 5 ml ddH_2_O using an ultrasonic bath and placed on a *LiChrolutRP-18*-column (Merck, Darmstadt, Germany [69]). The column was eluted with 2 ml ddH_2_O. The evaporated eluate was dissolved in 200 μl chloroform:*N,N*-diisopropylethylamine (1:1, v/v) followed by derivatization with 10 μl pentafluorobenzylbromide at 20°C overnight. After evaporation, samples were dissolved in 5 ml n-hexane and passed through a Chromabond-SiOH column (Machery-Nagel, Düren, Germany [70]). The pentafluorobenzyl esters were eluted with 7 ml n-hexane:diethylether (2:1, v/v). Eluate was evaporated, dissolved in 100 μl acetonitrile and analyzed by GC-MS as described by Miersch *et al*. [71]. All determinations were done using at least three independent biological replicates.

### Steady-state analysis of polar metabolites

After homogenization in liquid nitrogen, 80 ± 10 mg material was extracted by shaking with 360 μl methanol supplied with 6 μg ^13^C_6_-sorbitol (Sigma-Aldrich, Munich, Germany) at 70°C for 15 minutes. After addition of 200 μl chloroform, samples were shaken at 37°C for five minutes. Then 400 μl dH_2_O was added and samples were mixed by vortexing. A polar metabolite fraction enriched for primary metabolites and small secondary compounds was obtained after centrifugation at 16,000 *x* g for five minutes at room temperature. For backup and validation purposes, 80- and 160-μl aliquots of the upper polar phase were dried by vacuum centrifugation (Concentrator 5301; Eppendorf, Hamburg, Germany [72]). Chemical derivatization [73,74] and gas chromatography - electron impact ionization/time of flight - mass spectrometry (GC-EI/TOF-MS) metabolite profiling analysis [75,76] was performed essentially as described previously. GC-EI/TOF-MS chromatograms were visually controlled, baseline corrected and exported in NetCDF file format using ChromaTOF software (Version 4.22; LECO, [77]). GC-MS data processing into a standardized numerical data matrix and compound identification were performed using the TagFinder software [78,79]. Compounds were identified by mass spectral and retention index matching to the reference collection of the Golm metabolome database (GMD, [80-82] and to the mass spectra of the NIST08 database [83]). Guidelines for manually performed metabolite identification were the presence of at least three specific mass fragments per compound and a retention index deviation <1.0% [84].

All mass features of an experiment were normalized according to sample fresh weight, internal standard and maximum scaled. For quantification all mass features were evaluated for best specific, selective and quantitative representation of observed analytes. The Wilcoxon-Mann-Whitney test for each developmental stage was performed to identify metabolites that occurred in different amounts between WT and *jai1-1*. Correction of *P*-values for multiple testing by the Benjamini-Hochberg method was done using Multi-experiment viewer software MeV (Version 4.6.2; [85-87]. Metabolites were considered as differentially present when adjusted *P*-values were ≤0.05. Generation of groups was performed with Excel [88] using MAX normalized mean values of each metabolite and developmental stage. Grouping criteria were: developmental stage as depicted from the Venn diagram, direction of regulation and expression pattern in dependence on development. Down- and up-regulated metabolites were grouped according to their higher levels in WT and *jai1-1*, respectively.

### Determination of soluble sugar

Determination of soluble sugar contents was performed photometrically by a coupled enzymatic assay as described previously [89].

### Determination of water content, osmolytic values and anther dehiscence

Stamens were harvested and fresh weight (FW) was determined immediately. After drying at 50°C for one week, the dry weight (DW) was determined to calculate the water content (WC) according to the formula: WC = (FW-DW)/FW. To determine osmolytic values, stamens were harvested, frozen in liquid nitrogen and processed by several subsequently performed freeze-thaw steps followed by centrifugation at 17,000 × g for 30 minutes using a nylon sieve (41 μm) to collect the cell sap. At least 25 μl of cell sap were used directly for measurement of osmolytic values with a cryoscopic osmometer (Roebling, Berlin, Germany, [90]).

For determination of anther dehiscence, flowers and buds were harvested and sepals and petals were removed. Stamens were transferred inversely into a tube containing 200 μl phosphate-buffered saline and shaken for seven minutes. The number of released pollen was determined in technical triplicates using a counting chamber.

### Microscopy

For light microscopic analysis stamens were fixed in 3% (v/v) sodium cacodylate-buffered glutardialdehyde (pH 7.2), dehydrated in an ethanol series and embedded in epoxy resin [91]. Semi-thin sections (1 μm) were stained with toluidine blue. Freshly harvested stamens were sectioned into 200 μm thick cross sections using a vibrating blade microtome (VT 1000S; [92]). To evaluate starch accumulation, fresh sections were stained with one droplet of iodine potassium iodide solution for three to five minutes and washed with water.

Micrographs were taken using a Zeiss ‘AxioImager’ microscope (Zeiss, [93]) equipped with an AxioCam (Zeiss) and were processed through PHOTOSHOP 12.0.4 (Adobe Systems, [94]).

## Additional files

Additional file 1: Table S1. Tomato genes found in the microarray data to be differentially regulated in stamen of wild type and jai1-1 plants. **Table S2.** Max-normalized data of significantly accumulating metabolites obtained by steady-state analysis of soluble metabolites in stamen of wild type and *jai1-1* plants. Additional file 2: Figure S1. Detailed grouping of max-normalized differentially regulated genes according to their kinetics during development. **Figure S2.** Transcript accumulation of selected JA-responsive genes in stamens of wild type and *jai1-1* plants. **Figure S3.** Detailed grouping of max-normalized metabolites according to their kinetics during development. **Figure S4.** Accumulation profiles of selected metabolites identified by non-targeted metabolite profiling of stamens. **Figure S5.** Levels of soluble sugars in developing stamen of wild type and *jai1-1*. **Figure S6.** Morphology of stomia in stamen of stage 4. **Figure S7.** Pollen development in *NR* and *jai1-1 NR*. **Table S3.** Primer sequences.

## Abbreviations

ACC: 1-aminocyclopropane-1-carboxylic acid
AOC: ALLENE OXIDE CYCLASE
COI1: CORONATINE INSENSITIVE 1
*epi*: *epinastic*
ET: ethylene
GC-MS: gas chromatography-mass spectroscopy
JA: jasmonic acid
JA-Ile: JA isoleucine
*jail-1*: *jasmonic acid-insensitive-1*
JAZ: jasmonate ZIM domain protein
OPDA: cis-12-oxo-phytodienoic acid
*NR*: *Never Ripe*
RT-qPCR: reverse transcriptase quantitative polymerase chain reaction
TF: transcription factor
WT: wild type
