# Supplementary material for: Dissection of jasmonate functions in tomato stamen development by transcriptome and metabolome analyses
Source: BMC Biol. 2015 Apr 21;13:28. doi: 10.1186/s12915-015-0135-3 (PMC4443647; doi:10.1186/s12915-015-0135-3)
Supplement: Additional file 2: Figure S1. — Detailed grouping of max-normalized differentially regulated genes according to their kinetics during development. Figure S2. Transcript accumulation of selected JA-responsive genes in stamens of wild type and jai1-1 plants. Figure S3. Detailed grouping of max-normalized metabolites according to their kinetics during development. Figure S4. Accumulation profiles of selected metabolites identified by non-targeted metabolite profiling of stamens. Figure S5. Levels of soluble sugars in developing stamen of wild type and jai1-1. Figure S6. Morphology of stomia in stamen of stage 4. Figure S7. Pollen development in NR and jai1-1 NR. Table S3. Primer sequences. [file 12915_2015_135_MOESM2_ESM.doc]

**Additional file 2**


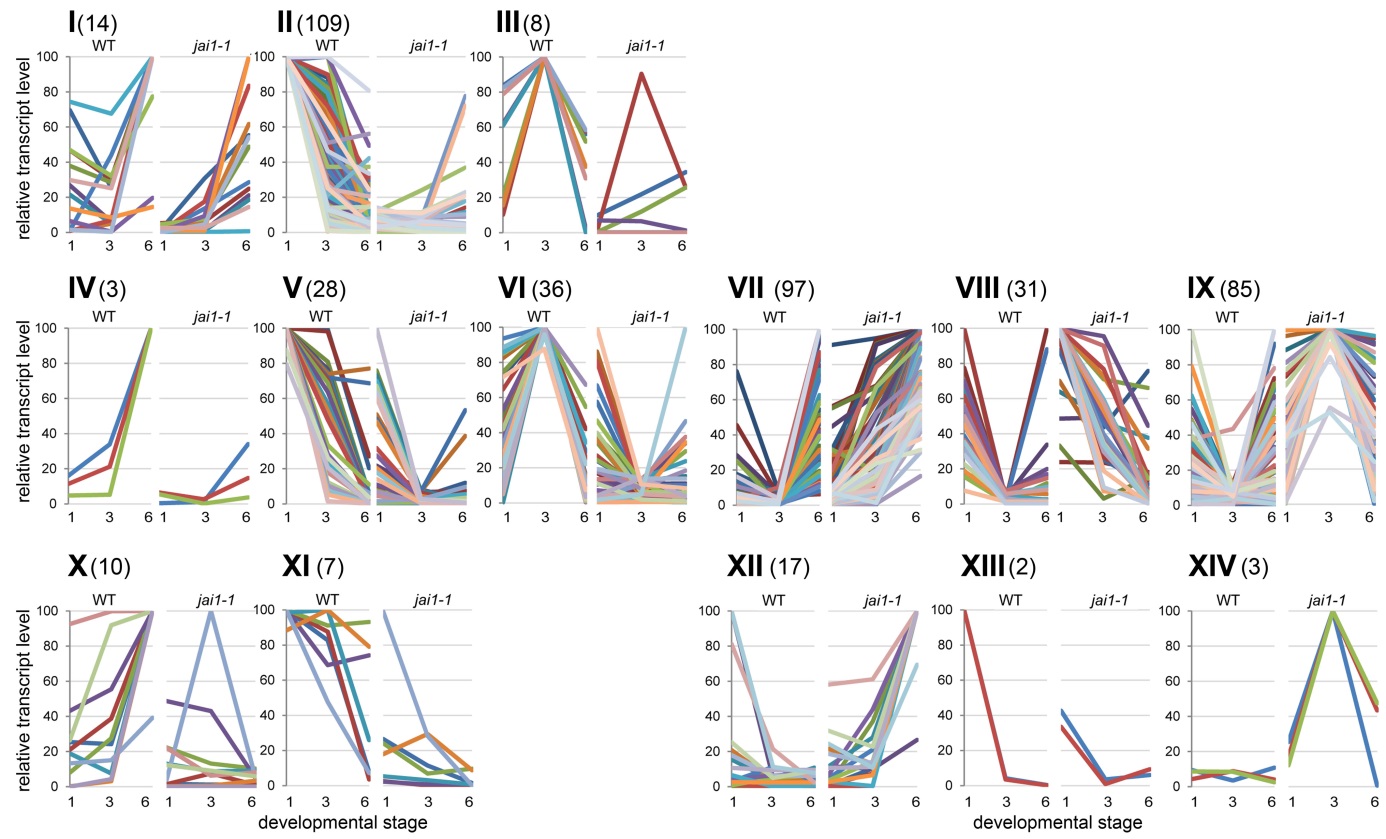


**Figure S1** **Detailed grouping of max-normalized differentially regulated genes according to their kinetics during development.**

Groups were generated by application of the following criteria: Stage-specificity according to the Venn diagram (I-III: 1, 1+3, 1+3+6, IV-IX: 3, X-XIV: 3+6, 6), higher expression levels in at least one developmental stage of wild type (I-VI, X-XI) or *jai1-1* (VII-IX, XII-XIV), and kinetics of transcript levels (I, IV, VII, XII: increasing during development, II, V, VIII, XI, XIII: decreasing during development, III, VI, IX, XIV: peaking at stage 3). The Arabic numbers show the number of differentially regulated genes in each group.


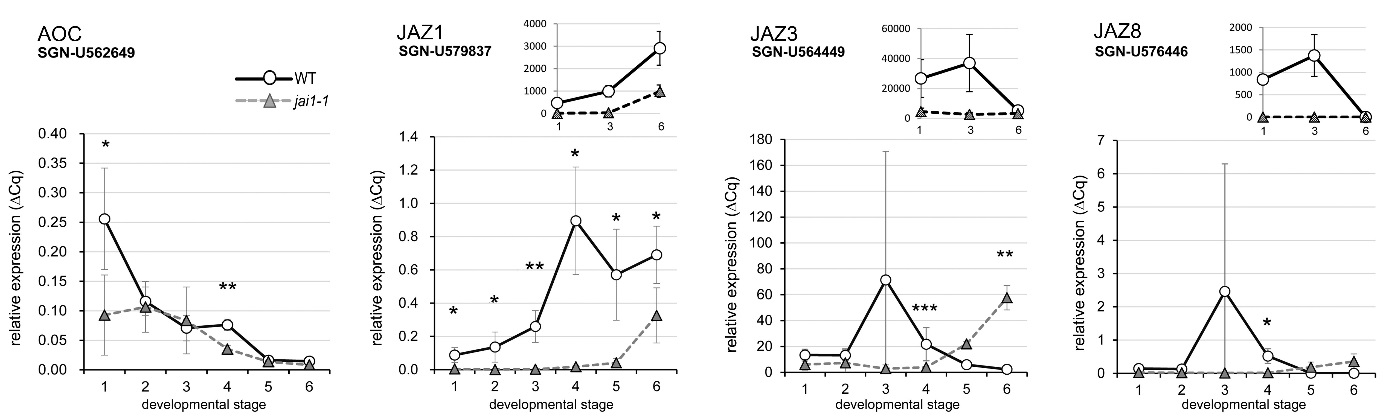


**Figure S2 Transcript accumulation of selected JA-responsive genes in stamens of wild type and *jai1-1* plants.**

Relative transcript levels of genes encoding early JA-induced genes were determined by RT-qPCR and set in relation to *SlTIP41*. The small diagrams on top visualize the signal intensities obtained from microarray analysis. Note that signal intensities of *SlAOC* were not deduced from microarray analyses, since this gene did not match the applied criteria for differential expression (at least 8-fold difference in expression in at least one developmental stage). Mean values ± SD are shown. Data of the same developmental stage were compared between wild type and *jai1-1* by Student’s t-test (* p ≤ 0.05, ** p ≤ 0.01, n = 3).


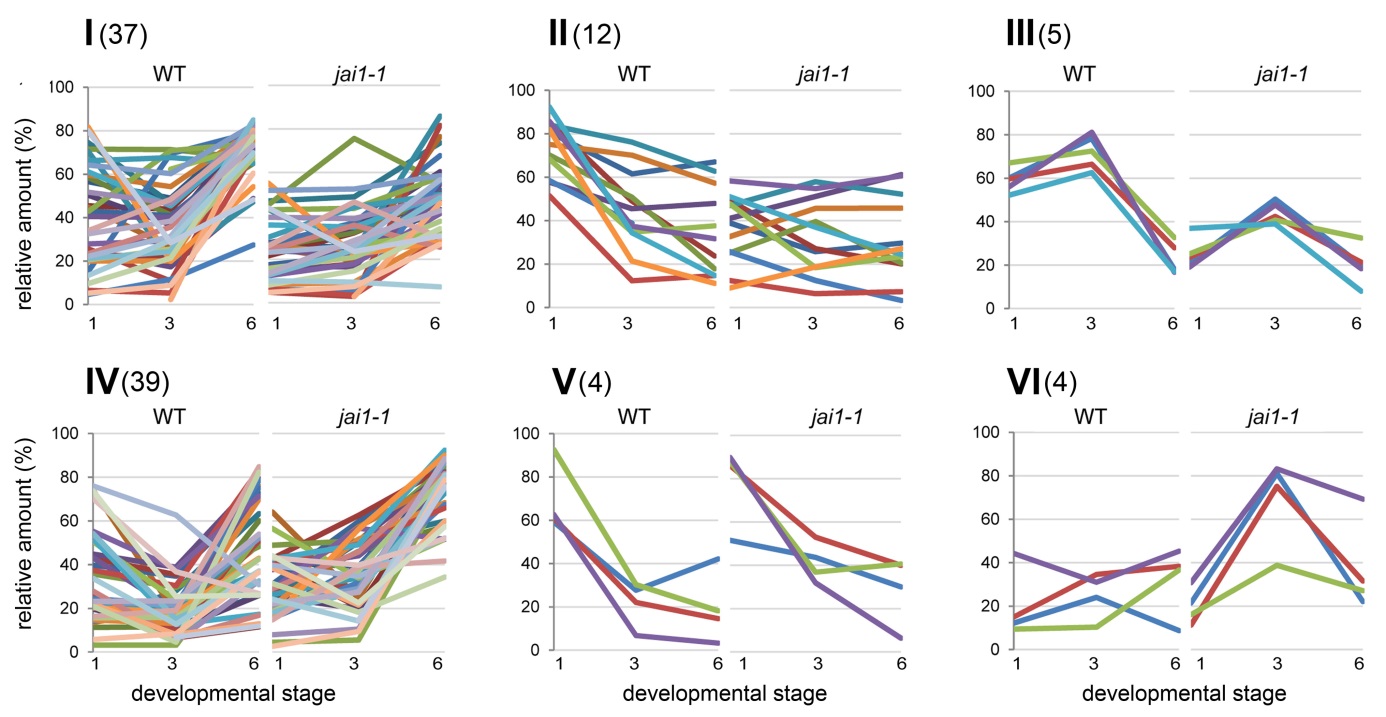


**Figure S3** **Detailed grouping of max-normalized metabolites according to their kinetics during development.**

Groups were generated by application of the following criteria: higher accumulation according to the Venn diagram (Figure 4A) in at least one developmental stage of wild type (I-III) or *jai1-1* (IV-VI), and kinetics of metabolite levels (I, IV: increasing during development; II, V: decreasing during development; III, VI: peaking at stage 3). The Arabic numbers show the number of metabolites in each group. Note that most metabolites show an accumulation during stamen development.


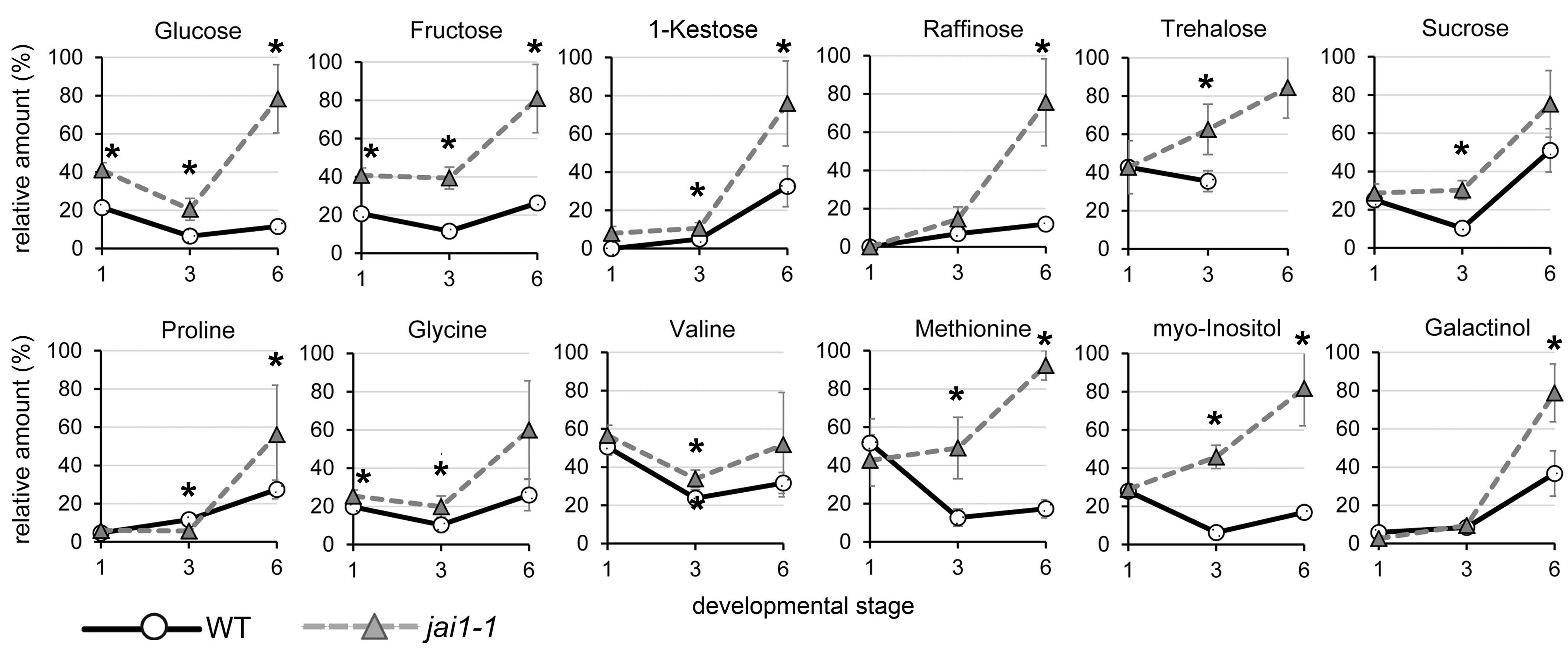


**Figure S4 Accumulation profiles of selected metabolites identified by non-targeted metabolite profiling of stamens.**

Relative (max-normalized) amounts of metabolites of group I (see Figure 5c) are shown. The selected metabolites exhibit significantly increased levels in stamen of *jai1-1* in comparison to wild type and an increase from stage 1 to stage 6. Data of the same developmental stage were compared between wild type and *jai1-1* by Student’s t-test (* p ≤ 0.05, n = 6).


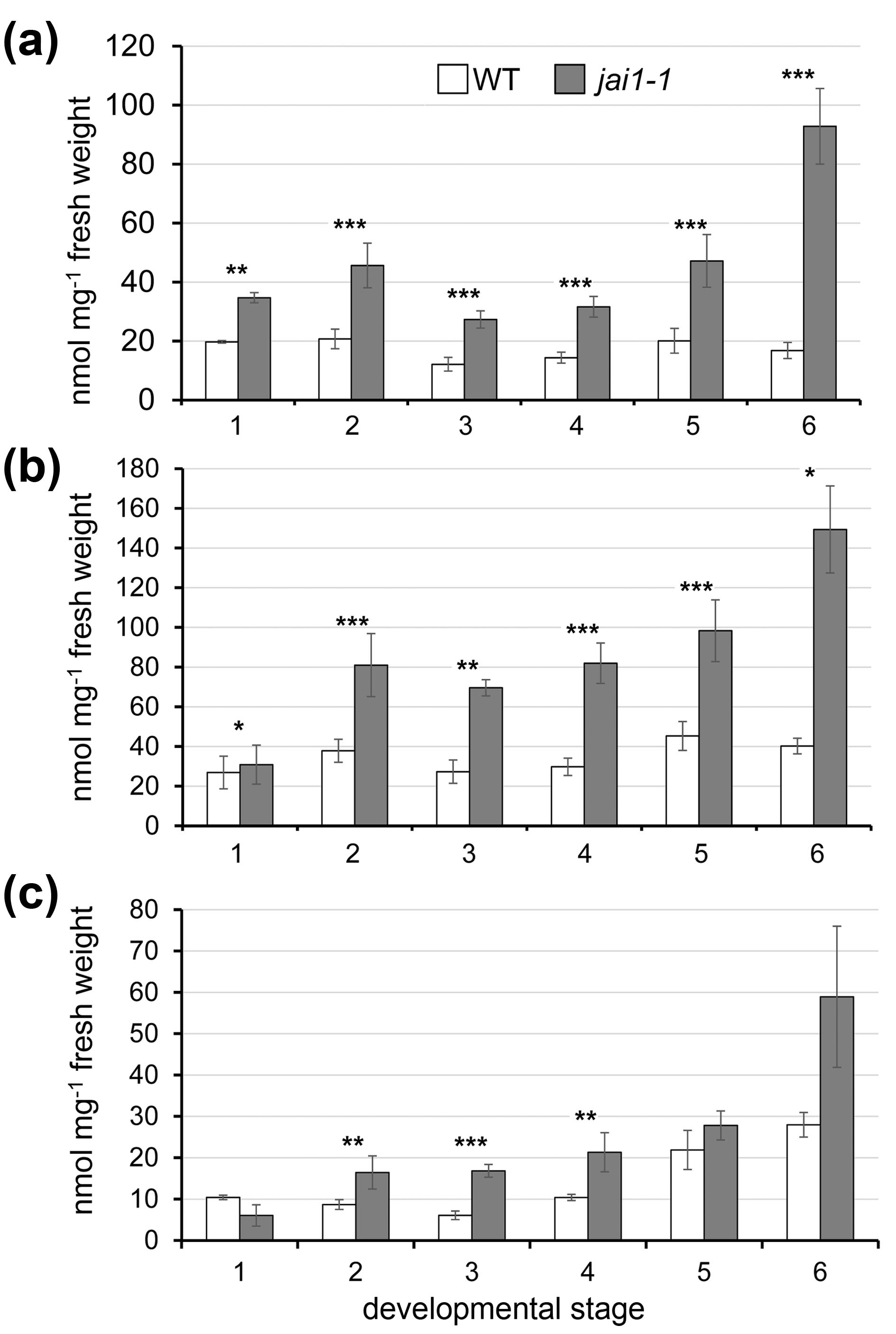


**Figure S5** **Levels of soluble sugars in developing stamen of wild type and *jai1-1*.**

Stamens of the respective stages were extracted and contents of glucose (**a**), fructose (**b**), and sucrose (**c**) were determined. Mean values ± SD are shown, * p ≤ 0.05, ** p ≤ 0.01, *** p ≤ 0.001 according to Student’s t-test (n ≥ 3).


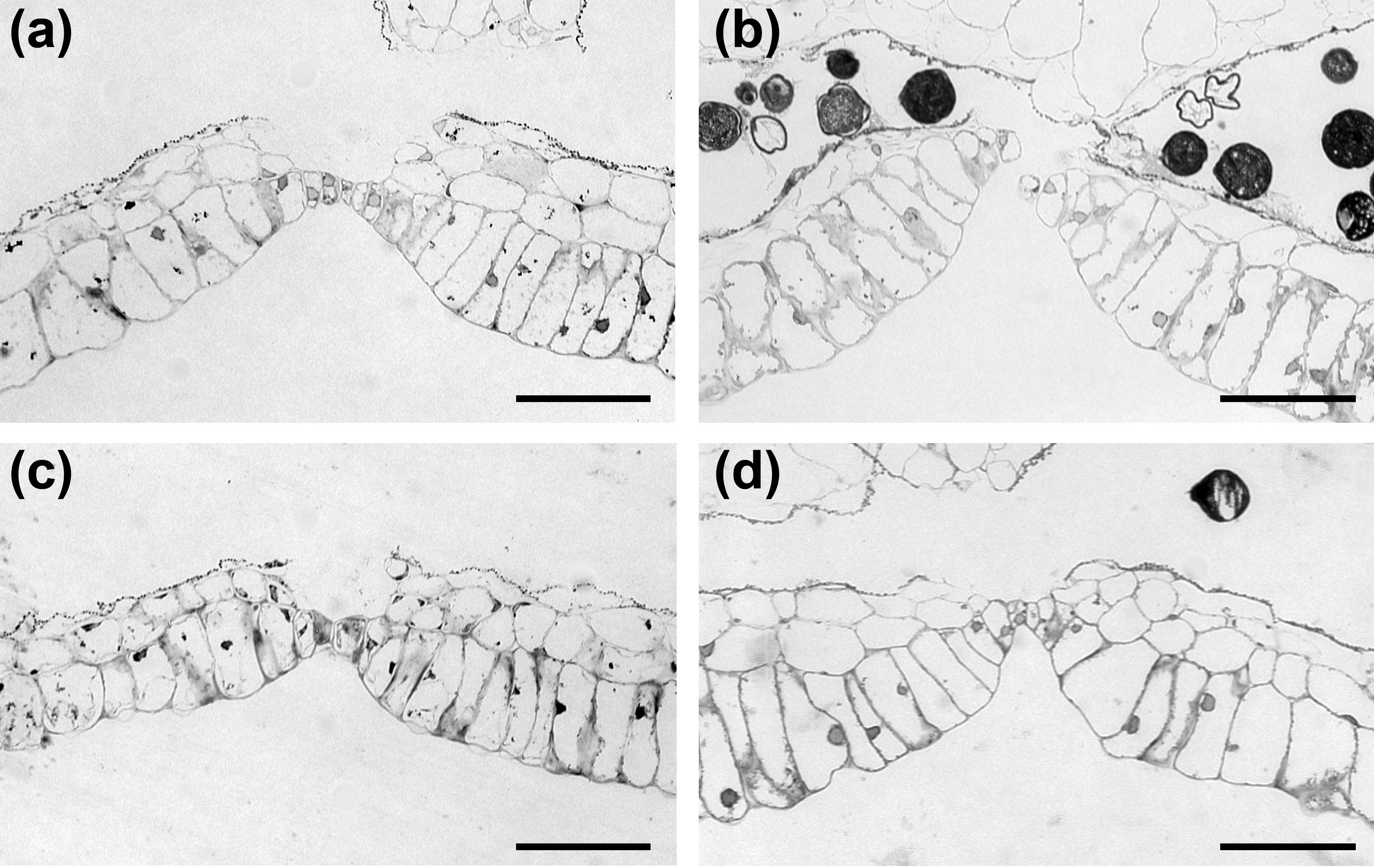


**Figure S6 Morphology of stomia in stamen of stage 4.**

Semi-thin cross-sections of the stomium region of anthers of wild type (**a**), *jai1-1* (**b**), *NR* (**c**) and the double mutant *jai1-1 NR* (**d**) stained with toluidine blue. Note that only in *jai1-1* the stomium is disrupted, whereas the other genotypes show a closed stomium. Bars represent 50 µm in all micrographs.


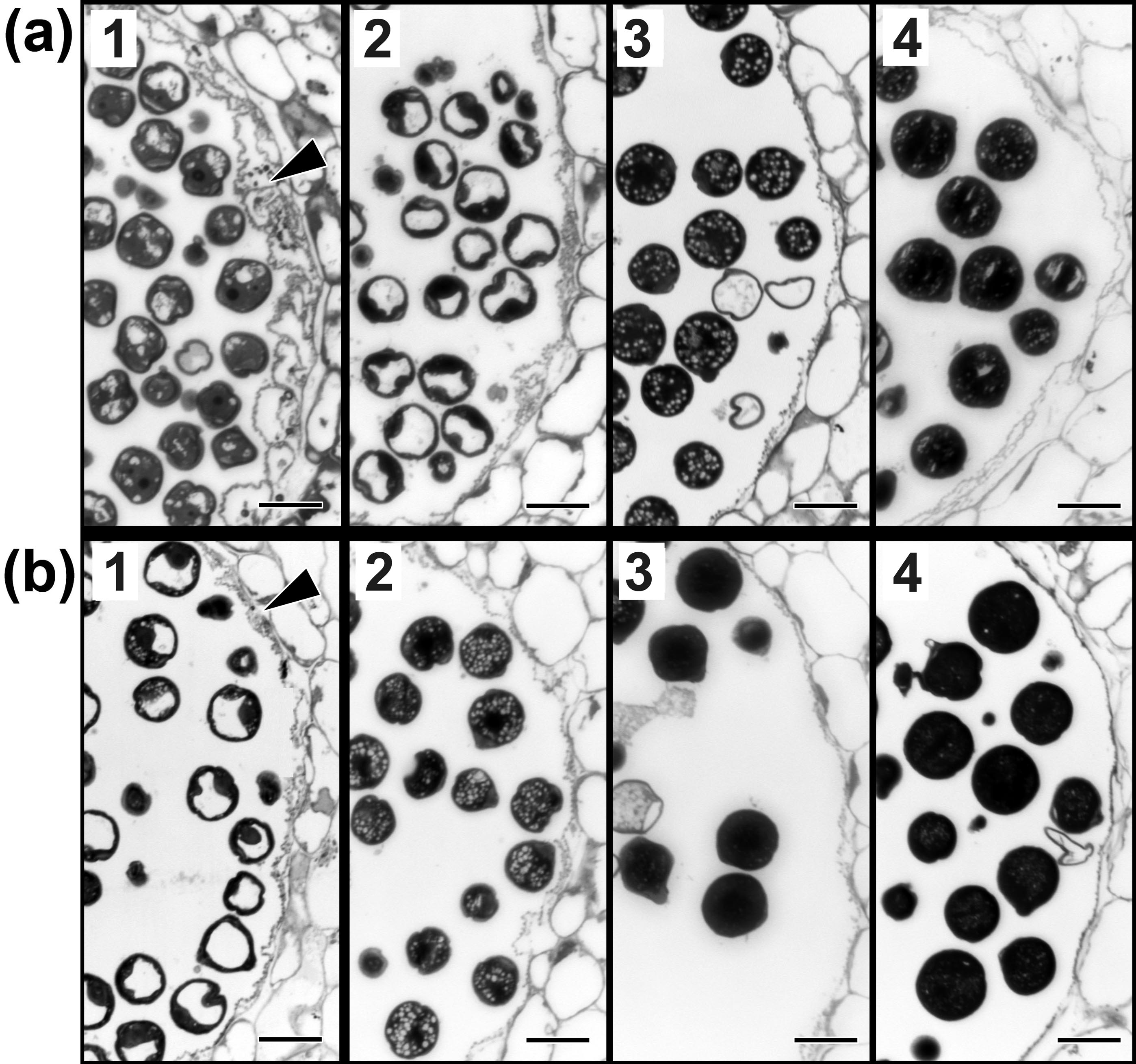


**Figure S7. Pollen development in *NR* and *jai1-1 NR*.**

Semi-thin cross-sections of anthers of the single mutant *NR* (**a**) and the double mutant *jai1-1 NR* (**b**) stained with toluidine blue. The developmental stages are indicated by numbers. Pollen of *jai1-1 NR* appear premature in development as visible by the big central vacuole in stage 1, presence of starch grains in stage 2 and the glassy state in stage 3. All these features appear in pollen of *NR* and wild type (see Figure 3) one stage later. Additionally, premature degeneration of tapetum (arrow heads) is visible in anthers of *jai1-1 NR*.

Bars represent 20 µm in all micrographs.

**Table S3: Primer sequences.**

| gene name | sequence [5'->3'] | | Tm [°C] | PCR product size [bp] |
| --- | --- | --- | --- | --- |
| (SGN-number) |
| ***jai1-1*-genotyping** | |  |  |  |
| WT | *for_* | CCA TGG AGT CCA TCA CCT AAC AGT | 62.7 | 525 |
| *jai1-1* | *for_* | GTG GTC AGA TCA GAG CCC TCT ATT | 62.7 | 777 |
| WT/*jai1-1* | *rev_* | GTG GAG ACG ATA TGT TGA GAC TAA | 59.3 |  |
| **reference gene (qRT-PCR)** | | | | |
| *TIP41* | *for_* | TCA GTG GGA GGA TTG TAA GG | 57.3 | 56 |
| (SGN-U584254) | *rev_* | GGT TCT TTA GAC GCC AAT GC | 57.3 |  |
| **ET-related genes** | | | | |
| *rinMADS* | *for_* | CAA ACA TCA TGG CAT TGT GG | 55.3 | 60 |
| (SGN-U578471) | *rev_* | ATG AGA AGG CTG TTC ATG TC | 55.3 |  |
| *fruitfull* | *for_* | GAA CCT TGC AGC TCA AAC TC | 57.3 | 56 |
| (SGN-U578128) | *rev_* | GGC AAA GGG TAA TTC CGA TG | 57.3 |  |
| *ASC8* | *for_* | GGG CGA TTT ACT CAA ACG AC | 57.3 | 45 |
| (SGN-U565888) | *rev_* | TTG TAG CCG CGG AGA CAA TG | 59.4 |  |
| *ACO* | *for_* | TCA GTG GTC TCC AAC TAC TC | 57.3 | 54 |
| (SGN-U577773) | *rev_* | GCA TCG GTG GAA CAT CAA TC | 57.3 |  |
| *AP2/ERF* | *for_* | CTT CTT TCA GCC ACA AAC TC | 55.3 | 52 |
| (SGN-U577093) | *rev_* | GCT GTT GCT ACT GCT ATT GC | 57.3 |  |
| *ETR6* | *for_* | GCT GCA GTG GTT GAA GAA TC | 57.3 | 53 |
| (SGN-U581694) | *rev_* | TTC TGT TCC GTC AAC CTG TC | 57.3 |  |
| *AOX1b* | *for_* | CGG GAC CTA AAT CAC TTT GC | 57.3 | 50 |
| (SGN-U589545) | *rev_* | AAC TCA TGC CCT TGA CAC TG | 57.3 |  |
| *Pirin-like* | *for_* | CCT GGA ATG CCT TCC TCT AC | 59.4 | 50 |
| (SGN-U574326) | *rev_* | AGA ACC AAA CGC TCC CTC TC | 59.4 |  |
| *Dehydrin* | *for_* | ACG CCA GCA TGG TAC TCT TG | 59.4 | 55 |
| (SGN-U290489) | *rev_* | GGA GCT AGA GCT GCC AGA AC | 61.4 |  |
| *Endoglucanase* | *for_* | GGA TGG CCC ATG GCT TAT TC | 59.4 | 48 |
| (SGN-U570620) | *rev_* | CAA TAG CAG CCC AAC TCA AC | 57.3 |  |
| **JA-related genes** | | | | |
| *AOC* | *for_* | TTC TAC TTC GGC GAT TAC GGT C | 60 | 50 |
| (SGN-U562649) | *rev_* | GGT TAA GTA CGC TCC CTG AAC G | 60 |  |
| *JAZ1* | *for_* | CTG ATC AAT CTG GTG TGA GTT TTG | 59.3 | 50 |
| (SGN-U579837) | *rev_* | CAG AAG GCT GTG GCA TTG AC | 59.4 |  |
| *JAZ3* | *for_* | CCC GAG TCT AAT GGA GTT GG | 59.4 | 56 |
| (SGN-U564449) | *rev_* | CTT ACC GGC TAA CAG AGG AG | 59.4 |  |
| *JAZ8* | *for_* | CAA GTA GAG GAA TGG AGA TG | 55.3 | 53 |
| (SGN-U576446) | *rev_* | ATG GTG ATG AAG GCT CAG AC | 57.3 |  |
